# Antimicrobial Agents Based on Metal Complexes: Present Situation and Future Prospects

**DOI:** 10.1155/2022/6819080

**Published:** 2022-12-08

**Authors:** Bharti Sharma, Sudeep Shukla, Rohit Rattan, Musarrat Fatima, Mayurika Goel, Mamta Bhat, Shruti Dutta, Rakesh Kumar Ranjan, Mamta Sharma

**Affiliations:** ^1^School of Biosciences and Biotechnology, BGSB University, Rajouri, Jammu and Kashmir 185234, India; ^2^Environment Pollution Analysis Lab, Bhiwadi, Alwar, Rajasthan 301019, India; ^3^WWF-India Field Office, ITI Road, Rajouri, Jammu and Kashmir 185132, India; ^4^Department of Botany, BGSB University, Rajouri, Jammu and Kashmir 185234, India; ^5^TERI Deakin Nanobiotechnology Centre, Sustainable Agriculture Program, The Energy and Resource Institute, Gurugram, Haryana, India; ^6^Amity School of Earth and Environmental Sciences, Amity University Haryana, Haryana, India; ^7^Department of Geology, Sikkim University, Gangtok, Sikkim, India; ^8^Aditi Mahavidyalaya, University of Delhi, New Delhi, India

## Abstract

The rise in antimicrobial resistance is a cause of serious concern since the ages. Therefore, a dire need to explore new antimicrobial entities that can combat against the increasing threat of antibiotic resistance is realized. Studies have shown that the activity of the strongest antibiotics has reduced drastically against many microbes such as microfungi and bacteria (Gram-positive and Gram-negative). A ray of hope, however, was witnessed in early 1940s with the development of new drug discovery and use of metal complexes as antibiotics. Many new metal-based drugs were developed from the metal complexes which are potentially active against a number of ailments such as cancer, malaria, and neurodegenerative diseases. Therefore, this review is an attempt to describe the present scenario and future development of metal complexes as antibiotics against wide array of microbes.

## 1. Introduction

Diseases caused by microbial pathogens are the major causes of morbidity and mortality throughout the globe. Each year, more than nine million deaths are attributed to the pathogenic disease worldwide [1]. Developing countries and underdeveloped countries have relatively more affected due to these diseases.

After the discovery of penicillin by Alexander Fleming in 1928, it was thought that humans had declared victory over microbes-based diseases. Since then, thousands of antibiotics have been consumed annually. WHO report on surveillance of antibiotic consumption (2016–18) states that “The overall consumption of antibiotics ranged from 4.4 to 64.4 defined daily doses (DDD) per 1000 inhabitants per day.”

However, just after Fleming's first use of penicillin for streptococcal meningitis in 1942, penicillin-resistant staphylococci were reported in hospitals and the community. The emergence of antibiotic resistance in microbes was observed; furthermore, microbes such as bacteria, fungus, bacteria, and parasites showed no response for the medicines, raising serious questions over human triumph over the microbial world. The antibiotic resistance in microbes not only has posed threat to human health but also for the agriculture and veterinary industries. Traditional antibiotics tend to follow the bullet-target concept, acting on specific biochemical processes: replication, transcription, translation, and other housekeeping metabolic enzymes, which provide ease of progressive resistance.

Ironically, the rate of development of new antibiotics is lagging behind the rate of emergence of antibiotics resistance; while, cost-effective global access to antibiotics is another challenge for health management systems in underdeveloped countries [2]. Metal and metal-based antimicrobial substances have great potential against antimicrobial resistance pathogens [3]. Metals are reported to target multiple cellular sites such as cellular membrane, genetic material, and reactive oxygen species-mediated cellular pleiotropic effects on microbial cells, contrary to organic antibiotics that act on specific targets on biochemical pathways such as replication, transcription, translation, and enzymatic reaction. Hence, there is an urgent need for the development of novel, wide spectrum antimicrobial agents that could target and eliminate antibiotics resistance microbes. A renewed interest in metals as antimicrobial and biocidal agents is reflected in hopes that less resistance will evolve.

## 2. Development of Antibiotics

Metals have been in use for their antimicrobial properties since thousands of years. The prevalence of Cu and Ag vessels and their use for water disinfection and food preservation since the time of the Persian Empire is well-known [4]. There are records of Paracelsus, a Swiss physician, using the silver internally and silver nitrate externally for treatment of wounds in the 1520s, which is followed even today. Similarly, silver sulfadiazine creams (Silvazine and Flamazine) are topical ointments that are used globally for the treatment of wound infections. Silver has also been used variedly in medicine, such as silver sutures for treating vaginal tears caused during the childbirth [5], silver nitrate (AgNO_3_) for preventing ophthalmia neonatorum [6], and silver foils for preventing surgical wounds from infection [7]. Moreover, compounds like Te, Mg, As oxides, Cu, and Hg salts have been used to cure diseases such as leprosy, tuberculosis, gonorrhoea, and syphilis [8–10]. This extensive use of metal based cures continued till the discovery of penicillin in 1920s. Now, as the humans are witnessing an ever-escalating threat of multidrug resistance, the use of antimicrobial metals is undergoing a much needed renaissance.

## 3. Metal Complex-Based Antimicrobial Compounds

Different metal complexes have their respective biological roles, and therefore, their design may help in developing new diagnostic probes as well as medicines. Metal complexes have emerged as great alternatives to organic compounds as they have specific steric and electronic effects that lead to different mechanisms of action (e.g., electron transfer and redox processes) [3].

Metals, because of being less electronegative, tend to promptly form positively charged ions, and this property lends them greater solubility in the biological fluids. The positively charged ions thus formed have affinity for electron-rich biomolecules, such as DNA and proteins, and play an important role in stabilizing/influencing their tertiary or quaternary structures. The current review shall revolve around the antibiotic compounds based on metal complexes.

### 3.1. Silver and Its Derivatives

Silver and its compounds have been in use as antimicrobial agents since ages. The antimicrobial properties of silver and its salts have been well researched and discussed [11–14].

**Figure 1:**
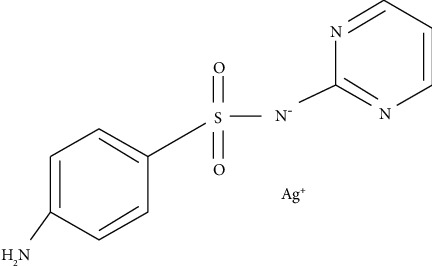
Silver sulfadiazine.

One important compound of silver is silver sulfadiazine (AgSD) (Figure 1). The antimicrobial activity of AgSD has been described by several authors [15–17].

Although silver and its complexes have shown cytotoxic effects against Gram-positive/Gram-negative bacteria and fungi, much is not known about the exact mechanism of action of silver except for its strong affinity to react with thiol (sulfhydryl, SH) groups in the bacterial cell, whether they be in structural or functional (enzymic) proteins. It has been demonstrated that silver induces structural changes in bacterial cells and interacts with nucleic acids [18, 19]. These interactions result in the denaturation of proteins further causing impairment of the membrane functions [20, 21]. Silver ions can produce ROS, which may target lipids, DNA, RNA, and proteins, and cause malfunctioning of membranes, proteins, and the DNA replication machinery [22, 23]. Moreover, DNA molecules in bacterial cytoplasm lose their ability to replicate upon treatment with silver leading to death of bacteria [20].

**Figure 2:**
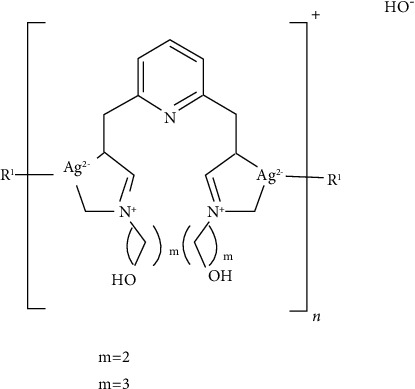
Ag NHC complex synthesized by youngs and coworkers.

Few newer categories of silver complexes such as N-heterocyclic carbene (NHC) complexes, phosphine complexes, or N-heterocyclic complexes of silver (I) have been found to have antimicrobial properties [24]. The NHC ligands make stable complexes and silver NHC complexes (Figure 2) help modulate release of silver for its systemic delivery. Various pincer Ag (I)-carbene complexes have exhibited antimicrobial activity against *Escherichia coli* (*E. coli*), *Staphylococcus aureus* (*S. aureus*), and *Pseudomonas aeruginosa* (*P. aeruginosa*), probably by intercalating with DNA or by disrupting the cell membrane [25].

A comparative study was conducted to understand antimicrobial activity of three tetrazole-containing compounds, 1-benzyl-1H-tetrazole (bntz), 1-benzyl-1H-tetrazol-5-amine (bntza), and 1-(4-methoxybenzyl)-1H-tetrazol-5-amine (mbntza) and silver (I) complexes of the general formula [Ag (NO3–O) (L-N4) 2]n, *L* = bntz (1), bntza (2), and mbntza (3). The study suggested that while silver (I) complexes 1–3 exhibited significant activity against a broad panel of Gram-positive and Gram-negative bacteria and fungi (with minimal inhibitory concentration values in the range 2–8 and 0.16–1.25 *μ*g/mL, respectively), the 1-benzyl-1H-tetrazoles used for the synthesis of the silver (I) complexes were not active against the bacterial and fungal strains. This pointed to the fact that the activity of these complexes was due to the presence of Ag (I) ion [26, 27]. Silver camphorimine complexes, obtained by reactions of mono or bicamphorimine derivatives with Ag (O) or Ag (Cl), demonstrated considerable activity against *Candida* species with oxo and hydroxo silver camphorimine complexes displaying particularly higher antifungal activity against *C. albicans* than *C. glabrata*.

Biologically synthesized silver nanoparticles (SNPs) are being widely using in the field of medicine. They have shown tremendous and very strong antibacterial properties against various bacterial species [28]. Savithramma et al. synthesized silver nanoparticles from stem bark extracts of *Boswellia* and *Shorea* and leaf extract of *Svensonia*. The SNPs synthesized from bark extracts of *Boswellia ovalifoliolata* and *Shorea tumbuggaia* showed toxic towards *Klebsiella* and *Aspergillus* and *Pseudomonas* and *Fusarium* species, respectively. But the AgSNP synthesized from leaf extract of *Svensonia hyderobadensis* exhibit strong effect against *Pseudomonas* and *Rhizopus* species [29].

Sondi and Salopek-Sondi studied that the concentration of Ag NPs plays an essential role to stop the growth of bacteria and the inhibition rate of *E. coli* is directly proportional to the concentration of Ag NPs. Ag NPs significantly damaged and destroyed cells and protein functions of *E. coli* due to its accumulation [30]. The antibacterial activity of Ag NPs is size dependent, since the small size Ag NPs (110 nm) have shown high tendency to interact with cell walls of bacteria [31].

### 3.2. Copper and Its Derivatives

**Figure 3:**
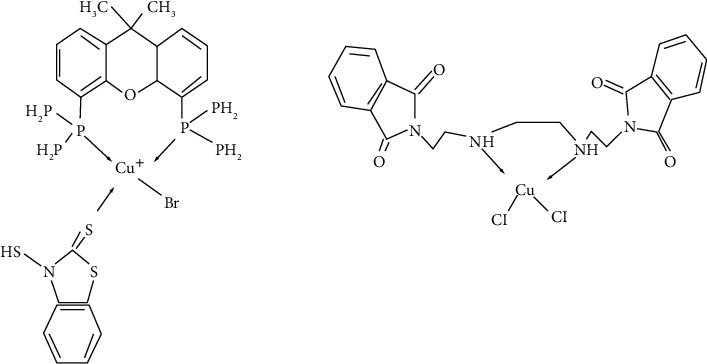
Structure of antimicrobial copper complexes (compound 4 and compound 5) [37, 38].

Copper is an essential metal needed by organisms for many functions but can be toxic in large concentration [32]. There are many copper-containing proteins present in microbes where copper acts as an electron donor/acceptor due to its ability to switch between copper (II) and copper (I) ions[33]. In order to improve its antimicrobial activity, many researchers studied the coordination of organic molecules with copper. There are different mechanisms of action that depend on the geometry of the complexes and the nature of the ligand (Figure 3) [34, 35]. Although the exact mechanism of the antimicrobial activity of copper is not known, many investigations have shown that reactive oxygen species (RoS) produced through Fenton-type reactions damages DNA. The release of copper ions causes inactivation of enzymes that leads to its toxicity [36].

**Figure 4:**
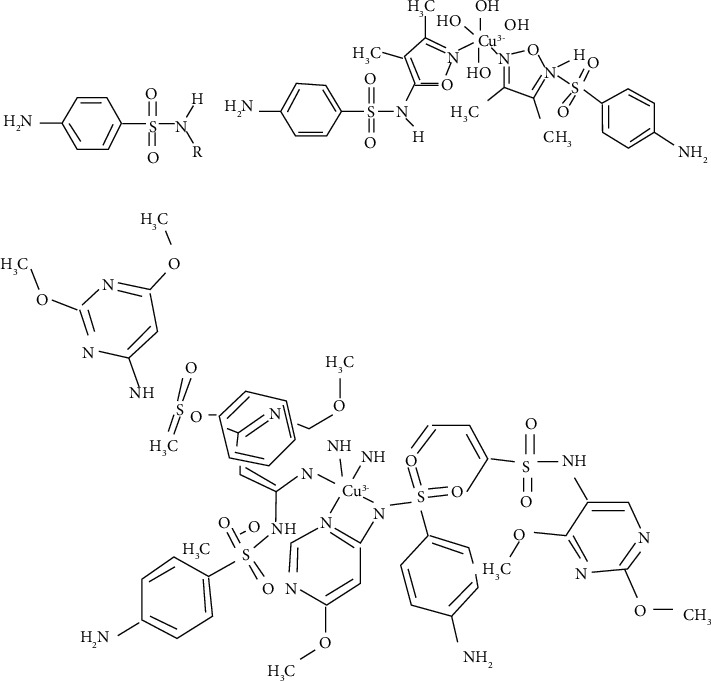
(a) General structure of sulfonamides; (b) structure of [Cu (sulfisoxazole) 2 (H2O)4 ]•2H2O7, the two oxazole rings and the copperion are in the same plane; (c) structure of a six-membered heterocycle substituted sulfonamide with its environment [33].

The compound 4, a tetrahedral mixed-ligand copper (I) bromide complex (Figure 4), exhibited 100 times greater activity against *Escherichia coli*, *Xanthomonas campestris*, *Bacillus subtilis* and *Bacillus cereus*, as compared to ampicillin [37] as it relied on disrupting the bacterial membrane by generating reactive oxygen species (ROS).

The antibacterial property of copper-based complexes is largely due to the formation of a phthalimide-based copper (II) complex 5 [38]. The phthalimide moieties and their derivatives are known to possess anticancer [39], antimicrobial [40], anti-inflammatory [41], and antimalarial properties [42], and this property stems from their capability to disrupt the DNA.

Sulfonamide ligands coordinated with copper (II) can interfere with the biosynthesis of tetrahydrofolic acid which is essential for bacterial metabolism [33, 43]. Studies have also revealed that copper (II) complexes with five-membered heterocyclic ring substituents (sulfisoxazole 7, sulfamethoxazole, and sulfamethizole) (Figure 3) have been found to possess greater antimicrobial activity against both Gram-positive and Gram-negative bacteria as compared to free sulfonamides [33].

Only the ionic form of free sulfonamides has an active antibacterial activity [44], but for its anionic form, the penetration efficiency across the lipoidal bacterial membrane is very low, which is due to its low lipophilicity. To enhance the permeation of the drug inside the cell, one possibility is to increase their lipophilicity by complexation of this kind of ligands with metal ions.

The antimicrobial activity of the deposited copper and copper oxide films against *Staphylococcus aureus* (*S. aureus*) and *Escherichia coli* (*E. coli*) was observed [45]. It was observed that there was 5-log_10_ reduction in the viable counts of *E. coli* on the copper thin films and 2-log_10_ reduction on copper oxide films after 30 minutes and 1 hour, respectively. But in case of *S. aureus*, both copper and copper oxide films exhibited 4-log_10_ reduction after 1 hour. The high antimicrobial efficacy of the Cu_2_O films, as compared to that of the pure copper films, suggests that oxide formation on copper objects should not significantly impair their antimicrobial activity. The novel Schiff base, ethyl 4-[ (E)-(2-hydroxy-4-methoxyphenyl)methylene-amino]benzoate (HL) and Six new copper(II) complexes, [Cu (L) (NO_3_) (H_2_O)_2_] (1), [Cu (L)_2_] (2), [Cu (L) (OAc)] (3), [Cu_2_ (L)_2_ Cl_2_ (H_2_O)_4_] (4), [Cu (L) (ClO_4_) (H_2_O)] (5), and [Cu_2_ (L_2_S) (ClO_4_) (H_2_O)] ClO_4_·H_2_O (6) were synthesized and characterized by using IR, UV-Vis, EPR, FAB mass spectroscopy, and molar electric conductivity [46]. Besides, the antimicrobial activity against *Escherichia coli* ATCC 25922, *Salmonella enteritidis*, *Staphylococcus aureus* ATCC 25923, *Enterococcus,* and *Candida albicans* strains was studied and compared with that of free ligand. It was observed that complexes 1, 2, and 5 showed a better antimicrobial activity than the Schiff base against the tested microorganisms.

Synthesis of copper nanoparticles using the modified polyol method was conducted to study its antimicrobial properties. The antimicrobial activity was studied against *Micrococcus luteus*, *Staphylococcus aureus*, *Escherichia coli*, *Klebsiella pneumoniae*, and *Pseudomonas aeruginosa* and fungus such as *Aspergillus flavus*, *Aspergillus niger*, and *Candida albicans*. The copper nanoparticles showed more inhibitory activity in bacteria than that in fungus [47]. In the last few decades, there has been significant progress in designing of different copper-based complexes having varied ligands, substituents, and geometries which exhibited significant antimicrobial properties.

### 3.3. Zinc and Its Derivatives

**Figure 5:**
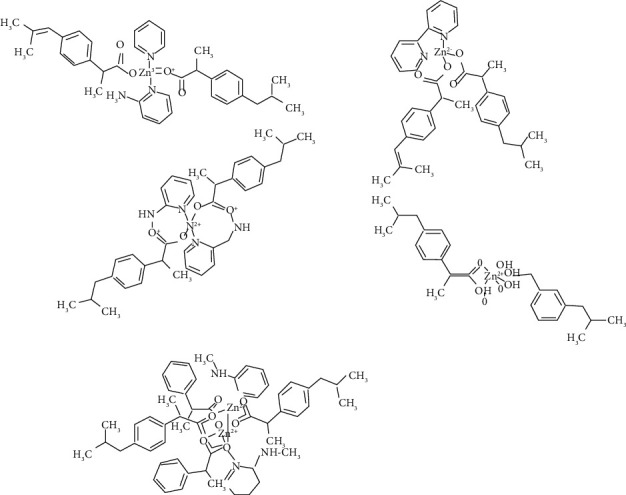
Structure of zinc-ibuprofen complexes [59].

It is an important element for living organisms as it is involved in many vital cellular reactions [48, 49]. Zn^2+^ ion plays a key role as metalloenzymes and in metal-based pharmaceuticals [50–52] especially as antiseptic [53]. Zinc inhibits the growth of many bacteria, e.g., *Escherichia coli*, *Streptococcus faecalis,* and some strains of soil bacteria [54]. There are two modes of its action: (i) direct action, whereby, microbial membrane is destabilized and its permeability is increased [55]; (ii) indirect action, whereby, interaction with nucleic acids leads to deactivation of respiratory enzymes [56]. The Zn (II) complexes have been found to exhibit antifungal activities against *Candida albicans* and *Aspergillus niger* which are around 4 and 10 times higher as compared to antifungal activities of fluconazole against them [57]. Most of the metal complexes have been found to exhibit greater activities as compared to the Schiff base ligand which are a result of the lipophilic nature of the complexes which eases cross-membrane movement. The square pyramidal Zn (II) complexes possess bacteriostatic as well as bactericidal properties against a wide array of bacterial and fungal strains [58]. A study was conducted to find out antimicrobial potential of Zn (II) complexes, involving ibuprofen with presence of N-donor heterocyclic ligands and with variable shapes and structures. The results revealed that antimicrobial activities of the complexes against Gram-positive (*Micrococcus luteus*, *Staphylococcus aureus,* and *Bacillus subtilis*) and Gram-negative (*Escherichia coli*, *Klebsiella pneumoniae,* and *Proteus mirabilis*) were significantly influenced by their geometries (Figure 5) [59].

**Figure 6:**
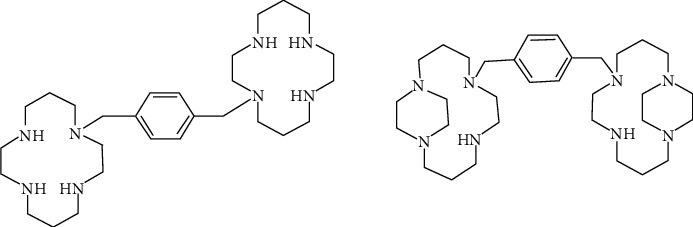
Antiviral macrocyclic bicyclams AMD3100 (9); constrained analogue of AMD3100 (10).

The complexation of cyclam to Zn (II) is another example that shows geometrical and structural importance of metallodrug. Some studies related to complexation of Xylyl-bicyclam, an anti-HIV drug to different types of metal ions, especially Zn^2+^, into the cyclam rings increases its coreceptor (CXCR4) binding strength which is the reason for its anti-HIV activity [60–62] (Figure 6, compound 9). Configurationally restricted analogue of bismacrocyclic cyclam CXCR4 receptor antagonist and its Zn (II) complex has been produced which lead to increased interaction with protein, therefore, resulted results in improvement of its anti-HIV activity [63] (Figure 6, compound 10).

### 3.4. Iron

Iron ion plays an important role in the growth of pathogenic bacteria; therefore, its coordination compounds with such organic molecules which display antimicrobial activity may be of great importance [64]. In this way, iron could be used as a carrier for the potent antimicrobial molecules, and the metalloantibiotics produced under this strategy would also enhance the efficiency of the antimicrobial drugs through efficient delivery. The iron-quinoxaline derivative compounds to cure tuberculosis were developed under this strategy which helped in greatly enhancing the antibacterial activity of quinoxaline derivatives. It was, therefore, concluded that such iron complexes have a significantly higher activity against *Mycobacterium tuberculosis* as compared to the free ligands [65, 66]. The higher activity of these iron complexes is the result of the fact that iron (III) efficiently carries the bioactive ligands and tends to increase their concentrations inside the target microbial cells. Similarly, iron (III) complexes of 1,2,4-triazole Schiff bases have also been revealed to exhibit greater antimicrobial action against various Gram-positive and Gram-negative bacteria as compared to their free ligands [67]. The presence of quinolones is the reason for the bioactivity of the complexes as they disrupt enzyme production. Chelation also helps increase the bioactivity of the complex as it enhances the lipophilic character of the central metal atom and helps it pass through the microbial membrane quite easily. There is potential of designing bio-organometallic derivatives with higher antimicrobial activity by including either an antimicrobial component, active moiety of the drug or the metal which resembles a part of the drug [68]. Organometallic complex ferroquine has been formed by adding a ferrocenyl moiety into the structure of the antimalarial chloroquine that results in better mode of action as compared to parent drug [69]. The ferrocene moiety present in the ferroquine turns it effective against even chloroquine-resistant strains as it produces reactive oxygen species that kill the parasites.

### 3.5. Ruthenium and Its Derivatives

**Figure 7:**
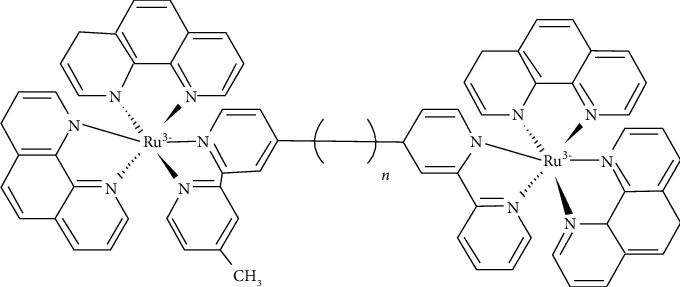
Structure of dinuclear ruthenium (II) complexes.

Ruthenium is the second member of group 8 transition metals (atomic number 44) and a potential antimicrobial agent. The octahedral ruthenium (II) complexes exhibit antimicrobial properties against *Mycobacterium smegmatis* [70]. While such ruthenium complexes have been reported to inhibit *M. smegmatis* at MIC of 2 *µ*g/mL, they have no effect on *S. aureus* (MSSA), *P. aeruginosa*, *E*. *coli*, *C. albicans*, and *C. neoformans*. Furthermore, the investigation of the antimicrobial activity of mono-, di-, and oligonuclear inert polypyridyl ruthenium (II) complexes is also deciphered [71]. It has been revealed that the dinuclear Ru (II) complexes linked by long flexible alkane chains (compound 18, Figure 7) exhibit significant inhibitory activity against both Gram-positive and Gram-negative bacteria but at the same time are less harming to human cells.

The nonsymmetric dinuclear polypyridyl ruthenium (II) complexes with one center of inert metal and other consisting of a coordinatively-labile metal, linked through bis [4 (4′-methyl-2,2′-bipyridyl)]-1,n-alkane ligand, exhibited significant activity against MRSA but comparatively were lesser effective against *E. coli* and exhibited almost no activity against *P. aeruginosa* [72]. The ruthenium (II) polypyridyl complexes with curcumin ligands exhibited good amount of inhibitory activity against drug-resistant*S. aureus* ATCC [73].

### 3.6. Gold and Its Derivatives

Gold has been used for the treatment of syphilis, tuberculosis, and inflammatory rheumatoid and also has great antimicrobial potential. Gold (I) alkynyl chromone complexes have been reported to have high levels of inhibitory activities against methicillin-sensitive (MSSA) and methicillin-resistant (MRSA) *S. aureus* but failed against *E. coli* [74]. Auranofin, a gold-based drug for the treatment of arthritis has been reported to inhibit thioredoxin reductase (Trx), an enzyme that helps bacteria in maintaining the thiol-redox balance and protects them against reactive oxidative species. The drug therefore exhibited significant activity against various Gram-positive bacteria including multidrug resistant bacteria as well as *M. tuberculosis*, but it has been ineffective against Gram-negative bacteria as the glutathione system in Gram-negative bacteria compensates for the loss of the reducing ability of Trx [75, 76]. In some cases, the outer membranes in Gram-negative bacteria have been found to be effective in avoiding auranofin accumulation [77]. The gold (I) bis-N-heterocyclic carbene complexes have exhibited notable activity against methicillin-resistant*S. aureus* (MRSA) strains and acted by disrupting the enzyme thioredoxin reductase (TrxRs) but were less effective when compared to auranofin or standard antibiotics [78].

### 3.7. Molybdenum and Its Derivatives

Several cis-dichloro/dibromodioxidobis (2-amino-6-substituted benzothiazole) molybdenum (VI) complexes have been found to exhibit inhibitory effects on bacterial species such as *P. aeruginosa*, *S*. *aureus,* and *K. pneumoniae* and fungi such as *A. flavus* and *A. niger*. The molybdenum (VI) complexes have exhibited inhibitory activity similar to ampicillin [79]. It has also been reported that isostructural 4,4-azopyridine (4,4′-azpy) pillared binuclear dioxomolybdenum (VI) complexes of formula ((MoO2L1) 2 (4,4′-azpy)], [(MoO2L2) 2 (4,4′-azpy)], and [(MoO2L3) 2(4,4′-azpy)] (where L# = Schiff base ligand) exhibited antimicrobial activities comparable to ampicillin and tetracycline at a concentration of 10 *µ*g per disc against *B. cerus* and *L. monocytogenes* (Gram-positive) and *E. coli* and *S. aureus* (Gram-negative) bacteria [80].

### 3.8. Aluminium and Its Derivatives

Aluminium oxide (Al_2_O_3_) nanoparticles (AlNPs) have diverse biomedical applications and favourable optical properties and a porous vast surface area. Due to their large surface area, AlNPs show strong antimicrobial activities. The antimicrobial activity of AINP was studied in *Escherichia coli*; they incubated 179 nm sized-AlNPs of various concentrations with *E. coli*. A mild antigrowth effect has been observed, which was due to the electrostatic interaction between the NPs and bacterial cells. Also, a small decrease was reported in extracellular protein content of the bacterium [81].

Aluminium oxide (AlNPs) and sulphur nanoparticles (SNPs) nanoparticles synthesized from *Colletotrichum* sp. have been studied for their inhibitory action against pathogens such as *Listeria monocytogenes*, *Salmonella typhi*, *Chromobacterium violaceum*, *Fusarium oxysporum*, and *Aspergillus flavus*, and it was found that while SNPs were most effective against *Salmonella typhi*, the AlNPs were significantly successful against *F. oxysporum*. It was also noted that the activity of several antibiotics also increased when used in combination with these metal-based nanoparticles. The synthesis and antimicrobial activity of aluminium (III) was reported [82]. The synthesis of N_2_O_2_ tetradentate Schiff base ligand from salicylaldehyde and o-phenylenediamine and the ligand reacted with Al (III). These Al (III) complexes show good antibacterial activity as compared to its ligands. The antimicrobial activity of the complexes is based on the chelation theory; chelation reduces the polarity of the metal atom because of partial sharing of its positive charge with the donor groups and possible *π*-electron delocalization within the whole chelate ring. Also, chelation increases the lipophilic nature of the central atom which subsequently favours its permeation through the lipid layer of the cell membrane [83].

### 3.9. Gallium and Its Derivatives

Ga can be used as an antimicrobial agent alone or can be combined with other materials. Ga can be used in several forms, such as Ga-protoporphyrin or Ga (III) tetra-(4-carboxypenyl) porphyrin (ClGaTCPP), for its antimicrobial activity. It was studied that the iron mimetic metal gallium Ga-protoporphyrin is recognised by the cell as iron, therefore, is metabolized via the same mechanism. This inhibits the essential pathways in bacterial cells, disrupts cellular respiration, and induces ROS production. Once Ga is digested, it disrupts vital cellular pathways (prevents electron transfer for ATP production by respiratory pathways, enzymes are inhibited to break down Ga, obstructing nutrient/iron release and promoting starvation, Ga's inability to be reduced such as iron blocks efflux pumps) [84]. Generally, this limits cellular respiration through the production of ROS, therefore damaging cell DNA and prompting cell death.

### 3.10. Indium and Its Derivatives

The antimicrobial activity of indium tin oxide (ITO) conjugated with T4 bacteriophage against *E. coli* was reported. It was observed that there was 99.9% reduction in bacterial concentration (*E. coli*) with bare as well as the amine, carboxylic, and methyl functionalized ITO/T4 surfaces. As anticipated, a single dose of immobilized bacteriophage was sufficient to eliminate further surge of bacterial population. All of the ITO/T4 systems maintained their antimicrobial activity in the presence of model food components. However, the antimicrobial activity was affected by pH; at pH 5, whereby, the bacteria's growth was physiologically inhibited, generally no reduction in *E. coli* concentration was detected [85].

The antibacterial activity of indium oxide thin film which is prepared using thermal evaporation of indium metal in vacuum on a glass substrate at 25°C and then subjected to thermal oxidation at temperature 400°C for 1 h was observed. In_2_O_3_ exhibited strong antimicrobial effects against Gram-negative bacteria. The results demonstrate that In_2_O_3_ causes damage to the bacterial cell membranes and controls the activity of some membranous enzymes which kills the *E. coli* and can be useful in the treatment of infectious diseases [86].

### 3.11. Mechanism of Action of Antimicrobial Metal Complexes

Various metals have been used in the treatment of different diseases; metals such as gold drugs, Myocrisin, and Auranofin are used for the treatment of rheumatoid arthritis. Their mode of actions is also different (Table 1). Silver and its complexes have shown cytotoxic effects against Gram-positive/Gram-negative bacteria and fungi, much is not known about the exact mechanism of action of silver except for its strong affinity to react with thiol (sulfhydryl, SH) groups in the bacterial cell, whether they be in structural or functional (enzymic) proteins. It has been demonstrated that silver induces structural changes in bacterial cells and interacts with nucleic acids [18, 19]. These interactions result in the denaturation of proteins further causing impairment of the membrane functions [20, 21]. Silver ions can produce ROS, which may target lipids, DNA, RNA and proteins, and cause malfunctioning of membranes, proteins, and the DNA replication machinery [22, 23]. Moreover, DNA molecules in bacterial cytoplasm lose their ability to replicate upon treatment with silver leading to the death of bacteria [20]. Copper has different mechanisms of action that depend on the geometry of the complexes and the nature of the ligand (Figure 3) [34, 35]. Although the exact mechanism of the antimicrobial activity of copper is not known, many investigations have shown that reactive oxygen species (RoS) produced through Fenton-type reactions damages DNA. The release of copper ions causes inactivation of enzymes that leads to its toxicity [36]. Zinc has two modes of mechanisms: (i) direct action, whereby, microbial membrane is destabilized, and its permeability is increased [55]; (ii) indirect action, whereby, interaction with nucleic acids leads to deactivation of respiratory enzymes [56]. The Zn (II) complexes have been found to exhibit antifungal activities against *Candida albicans* and *Aspergillus niger* which are around 4 and 10 times higher as compared to antifungal activities of fluconazole against them [57]. The antimicrobial activity of iron metal complex is due to the presence of quinolones, as they disrupt enzyme production. Chelation also helps increase the bioactivity of the iron metal complex as it enhances lipophilic character of the central metal atom and helps it pass through the microbial membrane quite easily. The possible mechanism of the bactericidal activity of polymeric ruthenium complex may involve a ROS dependent pathway. It is well-known that ROS such as superoxide anions (O_2_ • − ), hydrogen peroxide (H_2_O_2_), and hydroxyl radicals (OH•) damage lipids, proteins, and nucleic acids in cells, in a process that may lead to cell death [100, 101]. There is a similarity between Ga (III) and Fe (III) ions, and it is important that Ga (III) can substitute Fe (III) in iron-containing enzymes, thus repressing their activity [102]. The antimicrobial activity of Ga (III) is counteracted by an excess of Fe (III). Since many iron-containing enzymes are involved in critical functions in bacteria, such as DNA synthesis and repair, metabolism, respiration, and oxidative stress response [103], Ga (III) is likely to cause multiple deleterious effects to bacterial cells. Whether the antibacterial activity of Ga (III) relies on a general perturbation of bacterial iron metabolism or on the inhibition of a specific enzyme and/or cellular pathway remains an open question. The antimicrobial activity of the aluminium complexes is based on the chelation theory; chelation reduces the polarity of the metal atom because of partial sharing of its positive charge with the donor groups and possible *π*-electron delocalization within the whole chelate ring. Also, chelation increases the lipophilic nature of the central atom which subsequently favours its permeation through the lipid layer of the cell membrane [83]. It was proved that antibacterial activity of the Au nanoparticles is due to the attachment of these nanoparticles to the bacterial membrane followed by membrane potential modification and ATP level decrease and inhibition of tRNA binding to the ribosome [104].

## 4. Pharmaceutical Uses of Metal and Its Complexes

The metal complexes are nowadays used in the pharmaceutical industries against a number of diseases and are also acting as antimicrobial agents.

### 4.1. Antibacterial

Many antibiotics have been tested against many Gram-positive and negative bacteria yielding good results. As seen in case of ciprofloxacin, the antibacterial activity was enhanced by Zn; however, reverse was seen in amoxicillin and penicillin *G* because of inhibition of DNA gyrase after its penetration in the bacterial cell [105]. Another study showed that the enhanced lipophilicity of the metal complexes such as [cis, fac-RuCl_2_ (SO)_3_ (*μ*-nphen) cis, cis-RuCl_2_ (SO)_2_], [trans, mer-RuCl_2_ (SO)_3_ (*μ*-nphen) trans, cis-RuCl_2_ (SO)_2_], and [X]^+^[trans-RuCl_4_ (SO) (*μ*-nphen) mer-RuCl_3_ (SO)]^−^ showed antibacterial activity against *E. coli*. The increased lipophilicity of these metal complexes leads to breakdown of the permeability barrier thereby disturbing the respiration process in the bacterial cell [106]. Similarly, Co (II), Ni (II), Cu (II), Zn (II), Cd (II), and Hg (II) metal complexes with benzofuran derivative combines with Schiff bases, namely (E)-7-methoxy-N1-(2,4,5-trimethoxy benzylidene) benzofuran-2-carbohydrazide and (E)-N1-(2,6-dichloro benzylidene)-7-methoxy benzofuran-2-carbohydrazide which are reactive against *Staphylococcus aureus*, *Staphylococcus citreus*, *Bacillus polymyx*, *Bacillus cereus*, and *Lactobacillus* and Gram-negative species *Proteus mirabilis*, *Klebsiella pneumonia*, *E. coli*, *Salmonella typhi*, and *Pseudomonas aeruginosa*. Shiff's bases act as a chelating agent, and these heterocyclic rings of C=N bonds with the N_2_ and O_2_ donor system inhibit the enzymatic activity of bacteria [107]. The antibacterial activity is reported to be associated with the terpolymers of 2-amino-6-nitro-benzothiazole-ethylenediamine-formaldehyde against *Shigella sonnei*, *Escherichia coli*, *Klebsiella* species, *Staphylococcus aureus*, *Bacillus subtilis*, and *Salmonella typhimurium* [108].

Urea and its metal complexes after reacting with many metals resulted in formation of 1,3-diethyl-1,3-bis (4-nitrophenyl) which exhibits antibacterial activity against *Bacillus subtilis*, *Staphylococcus aureus*, *Escherichia coli,* and *Serratia marcescens*. The metallic complexes showed antibacterial activities and better inhibitory effects than ligand and standard drugs. This can be explained on the basis of Tweedy's chelation because of which the polarity of metal cation is lessened which is attributed to overlap of ligand orbital and partial sharing of positive charge of the metal ion. The chelation also enhances the delocalization of p-electrons over the chelate ring, thereby increasing the lipophilicity. This results in surging of penetration of complexes into lipid membranes, causing blockage of metal sites in enzymes of the target microbes. Also, the metal complexes inhibit the cell respiration and protein synthesis, thus affecting the growth of microorganism [109].

The two metal complexes of Cu (II), Zn (II), or Ag (I), namely, zeolite and synthetic zeolite showed antibacterial activity against *E. coli*. This is attributed to their potential of damaging DNA and altering enzyme activity because of increase in reactive oxygen species [110]. However, antibacterial activities against both Gram-positive and Gram-negative bacteria by graphene oxide are also observed. This results in damaging of cell membrane and growth inhibition by the oxidative stress, trapping microorganisms within GO sheets, cell membrane damage by sharpened edges of GO, and electron transfer interaction from microbial membrane to GO [111]. However, many metal complexes such as silver, copper, zinc, iron, ruthenium, gallium, bismuth, and vanadium are effective against either Gram-positive or Gram-negative bacteria, while some are effective against both by DNA intercalation [92].

Owing to the presence of the hydroxyl group, Schiff ligands have shown better antibacterial activity as compared to other groups against Gram-positive bacteria (*Bacillus subtilis* and *Staphylococcus aureus*) Gram-negative bacteria (*Escherichia coli, Serratia marcescens*, and *Pseudomonas aeruginosa*) [112]. Even the naturally occurring compound curcumin reacts with metal to form complexes and reactive against *Bacillus cereus*, *Bacillus subtilis*, *Staphylococcus aureus*, *Streptococcus mutans*, *Staphylococcus epidermidis*, *Escherichia coli*, *Pseudomonas aeruginosa*, *Yersinia enterocolitica*, and *Shigella dysenteriae* by the process of membrane disruption by inhibiting ATP-ase activity [88]. The antibacterial potential of metal complexes when combined with Schiff base is enhanced against *Bacillus cereus* and *E. coli*. This enhanced activity of the complexes may be attributed to chelation of Schiff base with metal ions that provide stability and more susceptibility against the bacterial pathogens [113]. Similarly, Schiff base (4-chloro-2-{(E)-[(4-fluorophenyl) imino] methyl} phenol) when reacting with metal (II) complexes (Mn (II), Co (II), Ni (II), Cu (II), and Zn (II)) show antibacterial activity against both Gram-positive bacteria such as *Bacillus subtilis* and *Staphylococcus typhi* and Gram-negative bacteria such as *Escherichia coli* and *Pseudomonas aeruginosa* [114]. However, bacteria such as *Staphylococcus aureus* LAC, *Streptococcus mutans*, and *Salmonella enterica* are affected by chlorhexidine-cyclamate [115].

Ruthenium complex ([Ru (X-phen) 2 (acac)]+^1^) binds to the bacterial surface of *Corynebacterium diphtheriae*, *Mycobacterium tuberculosis*, and *Staphylococcus aureus* and inhibit their growth by interfering in biological processes of growth inhibition by disturbing biological processes [35].

Besides, the addition of silver nanoparticles to the antibiotics has led to the enhanced antibacterial activity against *Staphylococcus aureus* and *Escherichia coli*. This is because of condensation of DNA molecules for the loss of its replication abilities and interaction of silver ions with thiol groups in protein, which causes inactivation of bacterial proteins [20, 116].

Different concentrations of silver nanoparticles exhibited antibacterial activity against Gram-positive bacteria irrespective of the pH, incubation temperature, incubation time by inhibited cell division and damaged the cell envelope, and cellular contents of the bacteria also be increasing bacterial cells size, and the cytoplasmic membrane, cytoplasmic contents, and outer cell layers exhibited structural abnormalities [117]. Moreover, tetracycline when combined with silver nanoparticles inhibits the growth of *Salmonella typhimurium*. This is because of the interaction of AgNPs to the bacterial cell wall, which leads to the alteration in membrane structure and enzyme activity [118].

### 4.2. Antifungal

Metal complexes by various inhibitory unique modes of action exhibit activity against many fungi. The coumarin complex and its metals such as copper, cobalt, nickel, and zinc exhibit antifungal activity against *Trichophyton longifusus, Candida albicans, Aspergillus flavus, Microsporum canis*, *Fusarium solani,* and *Candida glaberata*. The ligands with nitrogen and oxygen donor systems inhibit enzyme activity in fungi thus eradicating them [119]. Similar activity was also observed in sulfonamide which inhibits the growth of *Trichophyton longifusus, Candida albicans, Aspergillus flavus, Microsporum canis, Fusarium solani,* and *Candida glaberata* [120]. However, 4-methoxy2-amino thiazoles show antifungal activity against *Candida albicans* and *Aspergillus niger* because of inhibition of enzymatic activity in them [121]. Ketoconazole, miconazole, and clotrimazole metal complexes are known to possess antifungal activity by the inhibition of thioredoxin reductase enzyme [122]. Copper (II) 1,10-phenanthroline and 2,20-bipyridyl complex shows antifungal activity against *Candida albicans* and *Cryptococcus neoformans* by DNA cleavage activity and in silico molecular docking [123]. Copper and its compounds are effective against a wide range of fungi such as *Aspergillus carbonarius, Aspergillus fumigatus, Aspergillus niger, Aspergillus oryzae, Candida albicans, Cryptococcus neoformans, Epidermophyton floccosum, Microsporum canis, Myrothecium verrucaria*, *Saccharomyces cerevisiae*, *Torulopsis pintolopesii, Trichoderma viride, Trichophyton mentagrophytes,* and *Tricophyton rubrum* [124]. Copper, zinc, gallium, bismuth, and cobalt III based metal complexes show antifungal activity against many diverse fungi by eating up fungi [92]. Copper complexes act against *Candida albicans, Candida glabrata, Candida tropicalis, Candida parapsilosis,* and *Candida krusei* by damaging the cell membrane [125]. Metal complexes of peptides are used as antifungal agents by altering the DNA/RNA, protein and cell wall synthesis, permeabilization, and modulation of gradients of cellular membranes [126]. Metal complexes of cobalt when combined with Schiff's base display antifungal activity against *Candida* and *Cryptococcus* by degenerating the fungal hypha [127].

Complexes of copper and zinc ( (fluconazole), {[CuCl_2_ (fcz)_2_]^.^5H_2_O}_n_, and {[ZnCl_2_ (fluconazole)_2_]·2C_2_H_5_OH}_n_) shows antifungal activity against *Candida krusei* and *Candida parapsilosis* by disrupting the plasma membrane [128]. Silver complexes exhibit antifungal activity against *C. albicans* and *C. neoformans* by ligand exchange or release, ROS generation, redox activation, and catalytic generation of toxic species or depletion of essential substrates [127].

### 4.3. Antiprotozoan

Different metal complexes act against different parasites, protozoa being one of them. Giardiasis, leishmaniasis, malaria, trichomoniasis, and trypanosomiasis are some of the diseases against which new antibiotics drugs have been designed [129]. Antiprotozoan activity was exhibited by silver, copper, and chlorine metal complexes such as Mn (II), Co (II), Pt (II), and Cu (II) complexes. They showed an antiprotozoal effect and reduced the protozoal growth in water [130]. However, it also cannot be denied that annually many cases are reported about the increased infection in humans due to *Leishmania*. This can be attributed to nonavailability of antileishmania drugs or costly drugs, resistance, or other such reasons. So, metal complexes of Pt, Cu, Au, Ru, Bi, Tin are being used for drug designing as antileishmania [131]. However, metal complexes are also reported to be effective against *Hartmannella vermiformis* and *Naegleria fowleria* [124, 132]. So, research in this field is progressing as antiprotozoal drugs are being designed. Recently, three complexes, namely, [RuCl_3_ (trimethoprim) (1,4-bis (diphenylphosphino) butane)], [Cu (CH3COO)_2_ (trimethoprim)_2_], and [PtCl (trimethoprim) (triphenylphosphine)_2_] PF6 have shown pronounced antileishmania activity [133]. Trypanothione displays a unique pathway and trypanosomatid agent as trypanothione synthetase-amidase and trypanothione reductase enzymes are being designed to control the diseases caused by *Leishmania* by the residues of redox-active in cysteine and a histidine-glutamate couple (His461′- Glu466′) in trypanothione [134].

### 4.4. Antianthelmintic

An upsurge has also been witnessed with the use of antibiotics as antianthelmintic agents. The paralysing or killing of *Pheretima posthuma* was witnessed because of 10 different compounds of N-benzylidene pyridin-4-amines [135]. Also, the Schiff metal complexes of Co II, Cu II, and Ni II exhibited good results against *Pheretima posthuma* either by paralysing or killing the worm due to DNA cleavage [136]. 4-Aminoantipyrine, a Schiff base, is also known for its *in vivo* and *in vitro* anthelmintic properties [137]. Extract of silver nanoparticles prepared from silver complexes (silver nitrate) when mixed with extract of *M. charantia* indicated activity against *Pheretima posthuma* by the attraction of positive charge on the silver and the negative charge on cell membrane of microorganisms via electrostatic interaction [138].

## 5. Disadvantages of Metal Complexes

Metal complexes are a blessing for pharmaceutical industries, but on the other hand, the disadvantages they pose to the health of living organisms and to the environment cannot be ignored.

### 5.1. Cost Effectiveness

The bacterial infections arise frequently; therefore, the use of metal complex-based antibiotics is substantially higher in both the developing and the developed nations. For some indicators, the load of ailment is probably lower in one country as compared to others. Maybe at one place, the infection caused by one of the unique bacterial contaminations is probably low, also because of the fast length of the contamination; however, at other places, it can quite be the opposite. This may lead to continual conditions with a more widespread effect on the burden of disorder in developing nations than treatable/acute situations in the developed world. To optimise healthcare world over, it is desirable that policies of spending within reasonable limits be adopted. It also becomes important that new antibiotics are only prescribed when the price is moderate. Value-based pricing is a technique that can be used to decide a price for brand new antibacterial agents at which these drugs provide price for cash which ensures its affordability with uniformity in all the markets worldwide [139]. Moreover, quantifying the economic cost of antibiotics will require innovation within the use of current strategies to lay out studies that correctly gather relevant consequences and similarly research into new techniques for capturing broader monetary effects [140].

### 5.2. Emergence of Antimicrobial Strains

The emerging international problem of antimicrobial resistance has more than one aspect and involves resistance against many pathogens. More potent antibiotics, such as carbapenem and colistin, have grown to be a matter of terrific public health challenges [141]. One common subject matter is that antimicrobial drug use exerts selective stress favouring the emergence of resistance. Therefore, techniques to prevent the improvement and unfold of antimicrobial resistance depend upon the pathogens. Addressing antimicrobial use and resistance is one of the most urgent priorities in confronting rising infectious disease threats [142]. Those alarming threats are looking for the interest of the clinical community to increase newer antibiotics with long-lasting efficacy, least facet consequences, and occasional financial burden. For this reason, rigorous, well-designed, and well-dependent studies of exceptionally paramount importance to check the provision of more modern, surprisingly safe, and price effective antibiotics is required [141].

### 5.3. Environmental Perspective

Due to industrialization, heavy metals from industries move to the environment, resulting in severe environmental contamination. The modern agriculture techniques are also responsible for accumulation of heavy metal in the environment. The industrial wastes along with use of fertilizer, pesticides, weedicides, and herbicides cause adverse effects on all living things and their environment [143]. Air, water, and soil each and every part of earth is being contaminated by the heavy metals, thereby disrupting the food chain, causing health problems, and increasing mortality rate in living organisms including humans [144]. Even the elements exhibit speciation in the environment which is another matter of concern [145].

Cr, Ni, Cu, Zn, Cd, Pb, Hg, and As are toxic for the environment due to their accumulation and persistence. Chemicals such as heavy metals, dyes, pathogens, and fertilizers specifically cause pollution in water, which in turn imbalances the ecological life of humans and other organisms [146]. Water contamination is happening due to accumulation of heavy metals, dyes, and many other contaminants, which are toxic and carcinogenic for the biotic components [147]. In the case of increasing textile industries, not only quality of the waterbody is affected but an increase in the biochemical and chemical oxygen demand (BOD and COD) is seen. This largely affects photosynthesis, retards plant growth, enters the food chain, provides recalcitrance and bioaccumulation, toxicity, mutagenicity, and carcinogenicity in living organisms [148]. Cadmium is phytotoxic due to its high mobility in different trophic levels which in turn affect plant survival, reproductive success, and migration. And as a result, its diversity and genetic variety decreases [149].

Humans are also gathering antibiotics from the environment which is rendering negative effects on their own health [150]. They cause improper functioning of visceral organs, multiple sclerosis, Parkinson's disease, Alzheimer's disease, and muscular dystrophy [151]. Copper, zinc, cadmium, and lead are resulting in anaemia and because of which hypochromic and microcytic patterns are also seen in humans [152]. Cadmium causes respiratory, cardiovascular, and renal effects; chromium causes mental disturbance, cancer, ulcer, and hyperkeratosis; copper causes anaemia, and other toxicity effect includes indirectly through interaction with other nutrients: lead is neurotoxic, nickel causes skin allergies, lung fibrosis, diseases of cardiovascular system, and zinc causes abdominal pain, nausea, vomiting and diarrhoea, irritability, leathery, and anaemia in humans [153]. Besides, hyperpigmentation, keratosis, anaemia, neuropathy, and increased risk of developing several types of cancers in humans are because of these heavy metals [154]. Reactive oxygen species cause cancer. Apoptotic resistance causes cancer, and inflammation, epigenetic resistance affect methylation and acetylation. ARH-mediated effects cause serious cancer in humans and female puberty, and increased sensitivity of adipose tissue towards insulin, obesity, and neuronal developmental damage are caused by disruption of endocrine signaling [155, 156]. Heavy metal accumulation in the environment is a serious issue for the environment and health of living organisms; they cause serious diseases which cannot even be treated.

## 6. Future Prospects

The applications of metal complexes are still not developed much and, therefore, offers many opportunities in the coming time. Still, many basic principles for the novel synthesis, designing, and development of metal complexes for pharmaceutical purposes are inadequate. The burgeoning of many new processes and methods is expected to be helpful for the novel synthesis of the new compounds as therapeutic agents in the coming times.

However, by utilising diverse metal complexes, the underexplored chemical space for drug development can be addressed which opens options for testing of different metal complexes to predict their antimicrobial activity. This calls for deciphering the mechanism of the active compounds with different modes of action. It can be ascertained by finding out whether the metal complexes are inert which means the ligand stays intact as such but the whole rest of compound binds a specific bacterial target or partially liable, which means some ligands can get exchanged and produce a species that can binds itself to the microbe or is itself toxic or activity is fully mediated and ligand acts only as a carrier to deliver the metal ion to the target. Keeping in view of this, the mechanism of the different metal complexes also needs to be investigated so as to assess their action against different organisms in variable conditions. The selectivity, low toxicity, and in vivo stability of some heavy metals which gets activated in target or diseased tissue certainly makes metal complexes a better option over others, which can be explored, and hence, further improvements can be made for their use as antimicrobials. So, new research needs to be carried out in the coming times to explore new pharmaceutical potential associated with the metal-based complexes.

## 7. Conclusion

The translation of in vitro studies to in vivo experiments and subsequently to human scientific trials has been the primary mission in the development of new antimicrobial-based metal complexes. It, therefore, becomes vital to increase synthesis of such new metal complexes and to realize and understand their special modes of movement towards resistant pathogens. Combinational drug use can significantly deal with the problem, but even this combinational dose pattern may also cause resistance among pathogens. To triumph over the demanding situations of antibiotic resistance, antimicrobial compounds with a new mechanistic method need to be urgently sought. The future is vivid for this discipline of research, and in the upcoming years, it is expected that more metal complex-based antimicrobial compounds could not only be synthesized but be able to reach the medical trials and finally to the market.

## Figures and Tables

**Table 1:** Antimicrobial activity of some metals and their complexes along with their mode of action.

S. No.	Element	Complexes	Antimicrobial activity	Mode of action	References
s-block elements					
1	Lithium (Li)	LiC_6_H_7_O_6_	Lithium complexes are a good source of antioxidant	Increases the GABA level which in turn reduces glutamate and downregulates the NMDA receptors	[87]
2		C_4_H_6_LiNO_4_	Lithium complexes are a good source of antioxidant	Increases the GABA level which in turn reduces glutamate and downregulates the NMDA receptors	[87]
3		Li_2_CO_3_	Lithium complexes are a good source of antioxidant	Increases the GABA level which in turn reduces glutamate and downregulates the NMDA receptors	[87]
4	Calcium (Ca)	Ca (Cur)_2_	*P. verruculosum*, *A. niger*, *A. heteromorphus*, *A. flavus*, and *B. cereus*	Membrane disruption by inhibiting ATPase activity	[88]
p-block elements					
5	Gallium (Ga)	[GaCl_2_ (4- MepzH)_4_] GaCl_4_	Effective against HIV	Fe metabolism	[84]
6	Tin (Sn)	Sn (Cur)_2_	Have antifungal potential against *P. verruculosum*, *A. niger*, *A. heteromorphus*, *A. flavus,* and *B. cereus*	Membrane disruption by inhibiting ATPase activity	[88]
7	Lead (Pb)	Pb (Cur)_2_	Have antifungal potential against *P. verruculosum*, *A. niger*, *A. heteromorphus*, *A. flavus,* and *B. cereus*	Membrane disruption by inhibiting ATPase activity	[88]
d-block elements					
8	Cromium (Cr)	Cr (Curc)_3_	Shows antibacterial activity against *E. coli*, *K. pneumonia*, and *Pseudomonas* sp.	Membrane disruption by inhibiting ATPase activity	[88]
9		npapCr	Antibacterial activity against *P. aeruginosa*, *E. coli,* and *S. aureus* and antifungal activity against *A. flavus*, *C. albicans,* and *T. rubrum*. Also have antiviral potential against TMV and HSV.	Cytotoxicity	[89]
10	Manganese (Mn)	[MnL] Cl_2_	Antibacterial activity against *Salmonella typhi*, *Staphylococcus aureus, Escherichia coli*, and *Bacillus subtilis* and antifungal activity against *Aspergillus niger*, *Aspergillus flavus,* and *Rhizoctonia bataticola*	Disturbing respiratory mechanism and blocking metal binding site by delocalization of *π*-electrons over the whole chelate ring and enhances the penetration of the complexes into lipid membranes	[90]
11	Iron (Fe)	FeCur (OH)_2_	*E. coli* is the bacterial against which iron complex act	Membrane disruption by inhibiting ATPase activity	[88]
12		C_18_H_19_ClN_3_.C_5_H_5_.Fe	Antibacterial against *plasmodium falciparum*	Active against chloroquine-resistant parasitic strains by producing ROS *β*-lactamase	[89]
13		npapFe	Antibacterial activity against *P. aeruginosa*, *E. coli,* and *S. aureus* and antifungal activity against *A. flavus, C. albicans,* and *T. rubrum* and also have antiviral potential against TMV and HSV.	Cytotoxicity	[89]
14		[Fe (sulfamethoxazole)_2_ Cl_2_].2H_2_O	Antibacterial activity against *S. aureus*, *B. subtilis*, *P. aeruginosa*, *K. pneumonia*, and *E*. *coli*	Cytotoxicity	[43]
15	Cobalt (Co)	[CoCurCl] Cl	*Penicillium digitatum* fungi and bacteria such as *Streptococcus pyogenes*, *S. aureus*, and *A. flavus* against which antimicrobial activity is reported	Chain breakage	[88]
16		CoCurCl	*S. aureus*, *B. subtilis*, *S. typhi,* and *E. coli* are the bacteria against which cobalt complexes act.	Membrane disruption by inhibiting ATP-ase activity	[88]
17		[CoL] Cl_2_	Antibacterial activity against *Salmonella typhi*, *Staphylococcus aureus, Escherichia coli*, and *Bacillus subtilis* and antifungal activity against *Aspergillus niger, Aspergillus flavus*, and *Rhizoctonia bataticola* are reported	Disturbing respiratory mechanism and blocking metal binding site by delocalization of *π*-electrons over the whole chelate ring and enhancing the penetration of the complexes into lipid membranes	[89]
18		Co (sulfamethoxazole)_2_.3H_2_O	Possess antibacterial activity against *Mycobacterium tuberculosis*	Cytotoxicity	[43]
19		npapCo	Antibacterial activity against *P. aeruginosa*, *E. coli,* and *S. aureus* and antifungal activity against *A. flavus*, *C. albicans,* and *T. rubrum* and also have antiviral potential against TMV and HSV.	Cytotoxicity	[89]
20	Nickel (Ni)	[NiCurCl] Cl	*Penicillium digitatum*, fungi, and bacteria such as *Streptococcus pyogenes*, *S. aureus*, and *A. flavus* against which antimicrobial activity is reported	Membrane disruption by inhibiting ATPase activity	[88]
21		NiCurCl	*S. aureus*, *B. subtilis*, *S. typhi*, *P. aeruginosa*, and *E. coli* are the bacteria affected by nickel complexes		[88]
22		npapNi	Antibacterial activity against *P. aeruginosa*, *E. coli,* and *S. aureus* and antifungal activity against *A. flavus, C. albicans,* and *T. rubrum* and also have antiviral potential against TMV and HSV.	Cytotoxicity	[89]
23		Ni (sulfamethoxazole)_2_ Cl].2H_2_O	Antibacterial activity against *P. aeruginosa*, *Klebsiella pneumonia*, *B. subtilis*, *E. coli,* and *S. aureus*	Cytotoxicity	[43]
24		[NiL] Cl_2_	Antibacterial activity against *Salmonella typhi, Staphylococcus aureus, Escherichia coli*, and *B. subtilis* and antifungal activity against *Aspergillus niger, Aspergillus flavus*, and *Rhizoctonia bataticola*	Disturbing respiratory mechanism and blocking metal binding site by delocalization of *π*-electrons over the whole chelate ring and enhances the penetration of the complexes into lipid membranes	[89]
25	Copper (Cu)	[CuCl (H_2_itsc) (Ph_3_P)_2_] 2CH_3_CN	Possess antiviral activity and antitumor activity	Cell death	[91]
26		C32H16CuN8	*S. enterica* and *P. aeruginosa* bacteria for which antimicrobial activity was reported	DNA intercalation	[92]
27		[Cu (sulfisoxazole)_2_ (H_2_O)_4_] ·2H_2_O	*E. coli* and *S. aureus* bacteria for which antimicrobial activity was reported	Inhibiting folic acid synthesis	[88]
28		[CuCurCl] Cl	*Penicillium digitatum*, fungi, and bacteria such as *Streptococcus pyogenes*, *S. aureus* and *A. flavus* against which antimicrobial activity is reported	Membrane disruption by inhibiting ATPase activity	[88]
29		Cu (Cur)_2_	*S. aureus*, *E. coli*, *Klebsiella pneumonia,* and *Pseudomonas fluorescence*	Membrane disruption by inhibiting ATPase activity	[88]
30		CuCurCl	*S. typhi*, *P. aeruginosa*, and *E. coli* are effected by copper complexes	Membrane disruption by inhibiting ATPase activity	[88]
31		C32H16CuN8	Antibacterial activity shown against *S. enteric* and *P. aeruginosa*	DNA intercalation	[92]
32		[CuL] Cl_2_	Antibacterial activity against *Salmonella typhi, Staphylococcus aureus, Escherichia coli*, and *Bacillus subtilis* and antifungal activity against *Aspergillus niger, Aspergillus flavus,* and *Rhizoctonia bataticola*	Disturbing respiratory mechanism and blocking metal binding site by delocalization of *π*-electrons over the whole chelate ring and enhancing the penetration of the complexes into lipid membranes	[93]
33		Cu (sulfisoxazole)_2_ H_2_O	Antibacterial against *S. aureus* and *E. coli*	Cytotoxicity	[43]
34		Cu (sulfisoxazole)_2_ (H_2_O)_2_.3H_2_O	Antibacterial activity against *S. aureus* and *E. coli*	Cytotoxicity	[43]
35	Zinc (Zn)	[ZnCurCl] Cl	*Penicillium digitatum*, fungi, and bacteria such as *Streptococcus pyogenes*, *S. aureus*, and *A. flavus* against antimicrobial activity	Membrane disruption by inhibiting ATPase activity	[88]
36		[ZnL] Cl_2_	Antibacterial activity against *Salmonella typhi, Staphylococcus aureus, Escherichia coli*, and *Bacillus subtilis* and antifungal activity against *Aspergillus niger, Aspergillus flavus,* and *Rhizoctonia bataicola*	Disturbing respiratory mechanism and blocking metal binding site by delocalization of *π*-electrons over the whole chelate ring and enhancing the penetration of the complexes into lipid membranes	[94]
37	Ruthenium (Ru)	[Ru (Me_4_phen)_3_]^2^	Active against Gram-positive bacteria and *Mycobacterium tuberculosis*	Lipophilicity, charge, and charge separation conducted by Ru	[72]
38		[Ru (Me_4_phen)_2_ (acac)]^+^	Active against Gram-positive bacteria and *Mycobacterium tuberculosis*	Lipophilicity, charge, and charge separation conducted by Ru	[72]
39		[Ru (2,9-Me_2_phen)_2_ (dppz)]^2+^	Active against *S. aureus*	Lipophilicity, charge, and charge separation conducted by Ru	[72]
40		[Ru (dmob)_3_]^2+^	Active against *S. aureus*	Lipophilicity, charge, and charge separation conducted by Ru	[72]
41		([ru (X-phen)2 (acac)]+^1^	Antibacterial activity against *Corynebacterium diphtheriae*, *Mycobacterium tuberculosis*, and *Staphylococcus aureus*	Growth inhibition by disturbing biological processes	[35]
42	Palladium (Pd)	Pd (Curc)_2_	*E. coli* and *K. pneumonia* are the bacteria against which palladium act	Membrane disruption by inhibiting ATPase activity	[88]
43	Silver (Ag)	[HB (3,5- (CF3) 2pz)_3_] Ag (OSMe2)]	Have ability against *Staphylococcus aureus*	Cytotoxicity	[88]
44		Ag (I) carbene	Antibiotic for *E. coli*, *S. aureus,* and *P. aeruginosa*	Eat up bacteria	[92]
45		Ag (I)–saccharin complex	Antimicrobial activity against Gram + ve bacteria (*Micrococcus luteus* and *S. aureus*) and Gram-negative bacteria (*E. coli* and, *Proteus vulgaris*, and *P. aeruginosa*)	Eat up bacteria	[95]
46		Ag (I)-cyclamate	Antibacterial activity against *Mycobacterium tuberculosis, Mycobacterium avium, Mycobacterium intracellulare, Mycobacterium malmoense,* and *Mycobacterium kansasii*.	Eat up bacteria	[96]
47		Ag (I)-aspartame	Antibacterial activity against *Mycobacterium tuberculosis, Mycobacterium avium, Mycobacterium intracellulare, Mycobacterium malmoense,* and *Mycobacterium kansasii*.	Eat up bacteria	[96]
48		[HB (3,5-(CF3) 2pz) 3] Ag (thf)]	Have ability against *Staphylococcus aureus*	Cytotoxicity	[24, 97]
49		AgNO_3_	Effective against *Ophthalmia neonatorum*	Binding of free silver ions with tissue proteins, which leads to their precipitation and the obstruction of small vessels	[24]
50		C_10_H_9_AgN_4_O_2_S	Antibacterial activity against *Salmonella*, *E. coli,* and *S. aureus*	Cytotoxicity	[43]
51	Cadmium (Cd)	Cd (Cur)_2_	*Penicillium verruculosum, Aspergillus niger, Aspergillus heteromorphus, Aspergillus flavus,* and *B. cereus*	Membrane disruption by inhibiting ATPase activity	[88]
52		(CdL) Cl_2_	Antibacterial activity against *Salmonella typhi, Staphylococcus aureus, Escherichia coli*, and *Bacillus subtilis* and antifungal activity against *Aspergillus niger, Aspergillus flavus,* and *Rhizoctonia bataicola*	Disturbing respiratory mechanism and blocking metal binding site by delocalization of *π*-electrons over the whole chelate ring and enhances the penetration of the complexes into lipid membranes	[98]
53	Mercury (Hg)	Hg (Cur)_2_	*P. verruculosum*, *A. niger*, *A. heteromorphus*, *A. flavus,* and *B. cereus*	Membrane disruption by inhibiting ATPase activity	[88]
54		(HgL) Cl_2_	Antibacterial activity against *Salmonella typhi, Staphylococcus aureus, Escherichia coli,* and *Bacillus subtilis* and antifungal activity against *Aspergillus niger, Aspergillus flavus,* and *Rhizoctonia bataticola*	Disturbing respiratory mechanism and blocking metal binding site by delocalization of *π*-electrons over the whole chelate ring and enhancing the penetration of the complexes into lipid membranes	[99]
55	Vanadium (V)	[VOL] SO_4_	Antibacterial activity against *Salmonella typhi, Staphylococcus aureus, Escherichia coli,* and *Bacillus subtilis* and antifungal activity against *Aspergillus niger, Aspergillus flavus,* and *Rhizoctonia bataticola*	Disturbing respiratory mechanism and blocking metal binding site by delocalization of *π*-electrons over the whole chelate ring and enhancing the penetration of the complexes into lipid membranes	[1]

## Data Availability

No data were used to support this study.
